# An Active Peptide-Based Packaging System to Improve the Freshness and Safety of Fish Products: A Case Study

**DOI:** 10.3390/foods11030338

**Published:** 2022-01-25

**Authors:** Rosa Luisa Ambrosio, Marta Gogliettino, Bruna Agrillo, Yolande T. R. Proroga, Marco Balestrieri, Lorena Gratino, Daniela Cristiano, Gianna Palmieri, Aniello Anastasio

**Affiliations:** 1 Department of Veterinary Medicine and Animal Production, University of Naples Federico II, 80137 Napoli, Italy; rosaluisa.ambrosio@unina.it (R.L.A.); anastasi@unina.it (A.A.); 2 Institute of Biosciences and BioResources, National Research Council (IBBR-CNR), 80131 Napoli, Italy; marta.gogliettino@ibbr.cnr.it (M.G.); bruna.agrillo@ibbr.cnr.it (B.A.); marco.balestrieri@ibbr.cnr.it (M.B.); gratino.lorena@ibbr.cnr.it (L.G.); 3 Materias S.R.L., Corso N. Protopisani 70, 80146 Naples, Italy; 4 Department of Biology, University of Naples Federico II di Monte Sant’Angelo, Via Cintia 21, 80126 Naples, Italy; 5 Department of Food Microbiology, Istituto Zooprofilattico Sperimentale del Mezzogiorno, 80055 Portici, Italy; proroga.yolande@izsmportici.it (Y.T.R.P.); daniela.cristiano@izsmportici.it (D.C.)

**Keywords:** antimicrobial polymers, antimicrobial peptides, fresh fish, spoilage, fish quality, food safety, food packaging

## Abstract

Fresh fish are highly perishable, owing mainly to their moisture content, high amount of free amino acids and polyunsaturated fatty acids. Microorganisms and chemical reactions cause the spoilage, leading to loss in quality, human health risks and a market value reduction. Therefore, the fishing industry has always been willing to explore new technologies to increase quality and safety of fish products through a decrease of the microbiological and biochemical damage. In this context, antimicrobial active packaging is one such promising solution to meet consumer demands. The main objective of this study was to evaluate the effects of an active polypropylene-based packaging functionalized with the antimicrobial peptide 1018K6 on microbial growth, physicochemical properties and the sensory attributes of raw salmon fillets. The results showed that application of 1018K6-polypropylene strongly inhibited the microbial growth of both pathogenic and specific spoilage organisms (SSOs) on fish fillets after 7 days. Moreover, salmon also kept its freshness as per volatile chemical spoilage indices (CSIs) during storage. Similar results were obtained on hamburgers of *Sarda sarda* performing the same analyses. This work provides further evidence that 1018K6-polymers have good potential as antimicrobial packaging for application in the food market to enhance quality and preserve the sensorial properties of fish products.

## 1. Introduction

Today, health, nutrition and convenience are the major drivers in the global food industry. In this context, fish products have attracted considerable attention as a source of important nutritional components, such as high-quality protein, essential vitamins, minerals and polyunsaturated fatty acids (PUFA) [1,2]. Indeed, fish is considered of key importance for human nutrition all over the world, providing about 17% of the global intake of animal proteins [3]. However, its consumption in many parts of the world is far below the recommended level. As such, high-quality food with an extended shelf-life is essential for both producers and consumers. However, fish is a highly perishable product due to its relevant water activity, nearly neutral pH and specific composition that make it vulnerable to various biochemical, physical and microbial forms of deterioration throughout the production chain, thus causing rejection by consumers. Indeed, spoilage starts quickly after fish are caught, and rigor mortis is responsible for changes in fish after death.

Specifically, the degradation of various components and the formation of new products are accountable for the alterations in odour, flavour, colour and texture that happen during the spoilage process, so that deterioration occurs very rapidly due to mechanisms triggered by the microbial community, endogenous enzymatic activity (autolysis), and the chemical oxidation of lipids [4,5,6,7,8]. Due to all these changes, the shelf-life and quality of fresh fish are very limited, resulting in health risks as well as in enormous economic loss. Therefore, the fish industry is focused on preventing and controlling foodborne illness and microbial growth, which can lead to food spoilage, the major cause of fish loss, spoil estimated at millions of tons per year and accounting for 10% of the total production from capture fisheries and aquaculture [9]. These phenomena are even more evident in lower-income economies, in which spoilage and the quality downgrading of fish products occur due to high ambient temperatures, lack of infrastructure, basic technology and lack of cooling (cold chain) facilities. 

Salmon (*Salmo salar*) is one of the most consumed seafood in the world, either fresh or frozen, and a main product of aquaculture, accounting for 93% of total production [10]. Recently, its consumption has increased substantially because it offers several health benefits, mostly due to the presence of essential and vital nutrients for human body, such as omega-3 long-chain fatty acids. As this marine species has become a luxury product in the fishery market, any new strategy developed to enhance its safety and preserve its quality is considered essential for this economic sector. A considerable support in the fight against microbial spoilage may derive from food packaging, which can serve as a carrier of active substances, such as antimicrobials, playing an active role in food quality and shelf life, besides acting as a barrier against moisture, water vapor, gases and solutes. Specifically, antimicrobial packaging is considered a promising form of active packaging based on the immobilization of antimicrobial agents on the surfaces of polymers, thus providing antimicrobial properties to specific materials. 

In this context, polypropylene (PP) is an ideal food-safe thermoplastic material for packaging applications because of its low cost and its physical and chemical parameters. Therefore, it would be advantageous to impart antibacterial activity to these polymers, given the growing consumer interest in foods with fewer preservatives. In a previous study, the in-silico-designed antimicrobial peptide 1018K6, extensively characterized both from functional and structural points of view, was covalently bonded to commercial PET (polyethylene terephthalate) films and the ability of the developed antimicrobial packaging to improve the microbial quality and safety of dairy products was clearly demonstrated [11,12,13,14]. The applied procedure fulfilled the criteria of an efficient immobilization reaction, such as high yields and remarkable stability of the activated polymers, with no peptide release under different environmental conditions of use even after prolonged incubation times. Surprisingly, the AMP (antimicrobial peptide) was still active and preserved its excellent antimicrobial and antibiofilm abilities against a panel of Gram^+^ and Gram^-^ bacterial pathogens upon polymer surface functionalization, along with potent activity against moulds and fungal species, without exhibiting cytotoxic effects on human cells. Specifically, 1018K6 was able to explicate its bactericidal activity against both fungi, such as *Aspergillus brasiliensis,* the Gram^+^ pathogens *Listeria monocytogenes* and *Staphylococcus aureus* and the Gram^−^ *Salmonella* Typhimurium and *Escherichia coli* [15]. Furthermore, the optimized technology revealed the possibility of re-using the peptide polymers at least six times, while preserving its antimicrobial properties.

In this paper, 1018K6 was covalently immobilized onto PP surfaces, previously activated by plasma treatment, in order to extend the application field of our AMP-based system. Therefore, the impacts of prepared films on the physicochemical, microbial and sensory properties of fresh salmon fillets throughout storage at 4 °C for 7 days were investigated. A challenge test with *L. monocytogenes* was also performed. In order to validate the antimicrobial effectiveness of 1018K6-PP packaging in controlling the quality decay of fresh fish, the same analyses were performed on a different typology of fish-based foods, burgers of the bonito fish (*Sarda sarda*). Indeed, this typology of product has a softer texture with a lower shear force than other meat products reported in the literature, and it represents a valid solution for meeting consumer preferences for foods that have high nutritional value and are very convenient, being ready-to-cook. Moreover, it is well-known that the production phases of burgers are responsible for higher microbial concentrations than fillets due to the handling and the increased superficial area of the matrix for grinding. All these aspects outline the insidious profile of fish burgers and the challenge of testing the innovative packaging on these products.

## 2. Materials and Methods

### 2.1. Atmospheric Plasma Treatments

Openair-Plasma^®^ Technology was used as plasma treatment. The surfaces of commercial PP were cut into square-shaped pieces (4.0 × 4.0 cm dimension), which were cleaned with ethanol prior to use and then were placed on the plate at a distance of 3 cm to the nozzle. In all the treatments, air was used as the processing gas with a power of 440 watts and a speed of 10 mm/s. The effect of plasma on the polymer surfaces was evaluated by using the test Ink (Plasmatreat) in order to assess the wettability of the material.

### 2.2. Production of 1018K6

The peptide 1018K6 (VRLIVKVRIWRR-NH2) used in this work was purchased from GenScript Biotech (Leiden, Netherlands). It was stored as a lyophilized powder at −20 °C.

Analysis by mass spectrometry confirmed the identity of peptide.

### 2.3. 1018K6 Immobilization on Polymer Surfaces and Release Test 

Polymer surfaces pre-activated by atmospheric plasma treatment were incubated into solutions (3 mL) of 1018K6 prepared in distilled water at three concentrations (50, 100 and 200 μM) for about 4 h at 70 °C to fully remove the water. After drying, the functionalized PPs were immersed in a volume of distilled water equal to that evaporated for 16 h at room temperature in agitation, then they were sonicated for 20 min and the recovered solutions analysed by reverse-phase high-performance liquid chromatography (RP-HPLC) to indirectly estimate the immobilization yield. For these analyses, 200 μL of the samples were injected over a μBondapak C18 reverse-phase column (3.9 mm × 300 mm, Waters Corp., Milford, MA, USA) connected to a HPLC system (Shimadzu, Milan, Italy), using a linear gradient of 5–95% 0.1% TFA (Trifluoroacetic acid) in acetonitrile, at a flow rate of 1 mL/min. A reference solution was prepared with the initial peptide concentration used in the coupling reaction and was run in parallel. Therefore, by knowing the added peptide concentration (reference solution), the amount of peptide not covalently attached on the polymer surface was calculated by comparing the peak area and expressed as a percentage. A calibration curve of the C18 column using different 1018K6 concentrations was constructed. All measurements were performed in triplicate on three different preparations. 

To determine the stability of 1018K6 on the functionalized polymers, a release assay was performed by RP-HPLC using a linear gradient of 5–95% acetonitrile in 0.1% TFA, at a flow rate of 1 mL/min. A volume of 1 mL of pure water or NaCl 1 M was poured onto the functionalized polymers, which were incubated for 7 days at 4 °C, sonicated for 20 min and then the recovered solutions were loaded on RP-HPLC column. The solutions in contact with the functionalized polymers at time t = 0 were used as control samples and were run in parallel. All measurements were performed in triplicate on three different preparations.

### 2.4. Sample Preparation

Raw salmon (*Salmo salar*, Linnaeus 1758) from different batches were freshly bought from a local fishery industry (Naples, Italy). To evaluate the antimicrobial effects of the functionalized 1018K6-PPs polymers, two samples were prepared under aseptic conditions from each fillet that was sliced into pieces of approximatively 50 g. As a whole, the samples were separated into two groups: the control group (CTR-PP), including salmon fillets packaged in pre-activated PPs films not-functionalized with 1018K6 and the treated group, including salmon fillets packaged in PPs films functionalized with 1018K6 (1018K6-PP). Both 1018K6-PPs and non-functionalized PPs squares (4 × 4 cm) were placed on Petri dishes (both lid and base) in order to ensure constant contact between the pieces of salmon and the PPs. Subsequently, the packaged samples were refrigerated at 4 ± 1 °C for 7 days. The samples’ microbiological, physicochemical properties and quality aspects were analysed at days 0, 4 and 7.

Fish burgers of Atlantic bonito (*Sarda sarda*, Bloch 1793) were purchased from a fishing industry in Naples (Italy). A total of 21 burgers (200 g) were included in the experimental design. The samples were randomly divided into CTR-PP and 1018K6-PP groups and prepared as described above for the salmon samples. Once the fish burgers were packaged, the samples were stored at refrigeration temperature (4 ± 1°C) and sampled at days 0, 3, 5 and 7 to carry out the same analyses as were conducted on the salmon fillets.

The same analyses were performed also on fish samples packaged in PP films that were not subjected to any surface modification. 

### 2.5. pH and a_w_ Measurements 

The pH measurements were carried out with a digital pH meter (Crison-Micro TT 2022, Crison Instruments, Barcelona, Spain). Water activity (a_w_) was measured with Aqualab 4 TE (Decagon Devices Inc., Northeast Hopkins Court Pullman, Pullman, WA, USA). 

### 2.6. Microbiological Analyses 

Ten grams of each sample were added to 90 mL (1:10 *w*/*v*) of sterilized Peptone Water (PW, Oxoid, Madrid, Spain) in a sterile stomacher bag to be homogenized for three minutes at 230 rpm using a peristaltic homogenizer (BagMixer^®^400 P, Interscience, Saint Nom, France). Ten-fold serial dilutions of each homogenate were prepared. In order to better describe the microbial profile of samples and follow the growth trend of each bacterium responsible for the food alteration, the viable counts of various microorganisms were carried out. Total aerobic bacterial counts (TAB), both mesophilic and psychrophilic, were performed on plate count agar (PCA, Oxoid, Madrid, Spain) incubated at 30 °C for 48/72 h and 7 °C for 10 days, respectively (ISO 4833-1:2013 and ISO 17410:2019); total coliforms on violet red bile lactose agar (VRBL, Oxoid, Madrid, Spain) incubated at 37 °C for 48 h (ISO 4831:2006); Enterobacteriaceae on violet red bile glucose agar (VRBG, Oxoid, Madrid, Spain) incubated at 37 °C for 48 h (ISO 21528-2:2017); lactic acid bacteria (LAB) on MRS agar with Tween 80 (Oxoid, Madrid, Spain), incubated at 30 °C for 72 h (ISO 15214:2015); *Pseudomonas* spp. on pseudomonas agar base with CFC supplement (Oxoid, Madrid, Spain) incubated at 25 °C for 48 h (ISO 13720:2010); β-glucuronidase-positive *Escherichia coli* (ISO 16649-1:2018) on Triptone Bile X-glucoronide Agar (TBX, Oxoid, Madrid, Spain) at 44 °C for 24 h; *Brochothrix thermosphacta* on STAA (streptomycin thallous acetate actidione agar, Oxoid, Madrid, Spain) at 37 °C for 48 h; *Enterococcus faecalis* on KAA (kanamycin aesculin azide, Oxoid, Madrid, Spain) at 37 °C for 48 h; coagulase positive *staphylococci* on Baird-Parker agar (Oxoid, Madrid, Spain) at 37 °C for 24/48 h (ISO 6888-1:1999). After counting, the data were expressed as logarithms of the number of colony-forming units (CFU/g) and means and standard error were calculated. 

### 2.7. Challenge Test

Four fillets of approximately 150 g and from different batches were used to evaluate the inter-batch variability. All of them were tested in agreement with the AFNOR-BRD 07/10-04/05-Real Time PCR method in order to evaluate the absence of *L. monocytogenes* contamination. Three strains of *L. monocytogenes* isolated from fish samples were selected, following ISO 11290-1, to perform these analyses and stored in the Zooprophilactic Experimental Institute of Mezzogiorno biobank. All strains were re-suspended in diluent at 0.5 Mcfarland concentration, then a series of 10 times gradient dilution of *L. monocytogenes* was performed until to reach a concentration of 150 CFU/mL. All fillets were contaminated at surface to mimic contamination during the slicing, using one fillet not contaminated as a control. After artificial contamination, the samples were packed between two functionalized films and stored at 5 °C until 96 h. The enumeration of *L. monocytogenes* was performed according to Annex 1 of Reg (CE) 2073/2005 [16] at 24 h, 48 h, 72 h, and 96 h, in agreement with the reference methods EN ISO 11290-2. 

### 2.8. Colour

Colourimetric measurements of the surface appearance of salmon fillets and bonito fish burgers were performed using a Konica Minolta CR 300 colourimeter (Minolta, Osaka, Japan). The data were analysed in the CIELAB colour space, organizing in three orthogonal axes in a Cartesian coordinate system: lightness (L*), redness (a*) and yellowness (b*). Additionally, the angular coordinates of Hue angle [hab = ArcTan(b*/a*)], and chroma [Cab = (a*_2_ + b*_2_) ^1/2^] were calculated. Total colour difference (Δ*E*), variation in a* (Δa*) and in b* (Δb*) were calculated as:

$$\Delta E=\;\sqrt{{\left(L^{*}{}_{1}-L^{*}{}_{2}\right)}^{2}+{\left(a^{*}{}_{1}-a^{*}{}_{2}\right)}^{2}+{\left(b^{*}{}_{1}-b^{*}{}_{2}\right)}^{2}}$$

$$\Delta a^{*}=a^{*}{}_{2}-a^{*}{}_{1}$$

$$\Delta b^{*}=b^{*}{}_{2}-b^{*}{}_{1}\;$$

where L*_2_, a*_2_, and b*_2_ are the values recorded at a specific day during the storage; instead, L*_1_, a*_1_, and b*_1_ are values collected at day 0.

Δ*E* represents the result of changes in lightness (ΔL*), redness (Δa*) and yellowness (Δb*), which do not always change in parallel. For this reason, Δa* and Δb* were taken into account. Since the colour may not be homogeneous over the entire surface of fillets and burgers, five superficial measurements were performed for each sample to obtain representative results.

### 2.9. TBARS, Total Volatile Basic Nitrogen (TVB-N) and Trimethylamine (TMA) Analyses

Lipid oxidation was monitored by determining the thiobarbituric acid (C_4_H_4_N_2_O_2_S) substances expressed as malondialdehyde (CH_2_(CHO)_2_) concentration (mg/Kg), which represent secondary oxidation products. Measurements were performed according to the method proposed by Ambrosio et al. [17].

The TVB-N and TMA values for all salmon and fish burger samples were quantified according to Conway’s micro-diffusion method [18]. The results were expressed in mg of nitrogen per 100 g of sample.

### 2.10. Sensory Testing

Sensory testing of salmon fillets and bonito fish burgers was undertaken by a panel consisting of five trained panellists. The judge’s acceptability study was assessed through a sensory evaluation, taking into account odour, colour and general acceptability. Appropriate attributes have been fixed in order to minimize individual differences and ensure the results’ repeatability. Sensory assessments were performed under controlled humidity, light and temperature. A Likert scale (9-point) was used to assess each attribute; in the scale, 9 corresponded to excellent, 8 to very good, 7 to good, 6 to reasonable, 5 to not good (acceptable limit), 4 to disliked, 3 to bad, 2 to very bad, and 1 to completely unacceptable [19]. Coded samples were randomly and simultaneously distributed to each panellist. 

### 2.11. Statistical Analyses

Physicochemical and microbiological data were statistically analysed with generalized linear mixed model of SPSS version 26 (IBM Analytics, Armonk, NY, USA). Analysis of variance was performed to study parameters of salmon fillets and bonito fish burgers at each sampling time, including the fixed effect of packaging used and storage times. An a posteriori contrast was carried out using the Tukey test, considering a *p* value of <0.05 as statistically significant. 

## 3. Results

### 3.1. 1018K6 Immobilization on PP Surface

Following confirmation of the excellent antimicrobial properties preserved by 1018K6, even after bonding on different materials, such as PET and nanoparticles [13,20], the peptide was further immobilized on another plastic polymer commonly used in food packaging, polypropylene (PP).

To this aim, commercial PP slides were exposed to plasma treatment to activate the inert polymeric surfaces with reactive -COOH* functional groups that are available to interact with the amine moieties of 1018K6, forming amide bonds [21]. 

**Figure 1:**
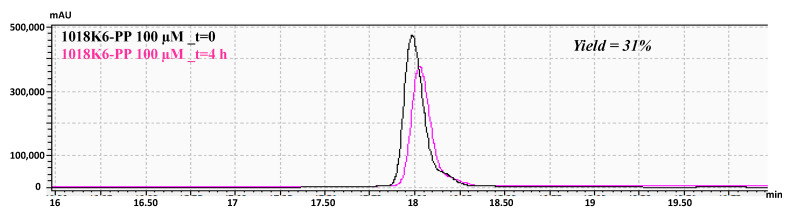
Immobilization yield (%) of 1018K6 on PP surfaces determined by RP-HPLC chromatography on a C18 column after the coupling reaction. PP surfaces pre-activated by plasma treatment, were incubated with a water solution of 1018K6 (100 μM). After the coupling reactions, the supernatants were recovered and analysed by RP-HPLC. The peptide solution (100 μM) at time 0 (t = 0) was used as control. The reported chromatograms are representative of three independent experiments.

In order to develop an antimicrobial packaging more adequate for food application, the conditions applied in our previous studies to functionalize the polymeric materials were modified. Specifically, the covalent attachment of 1018K6 on the pre-activated PP polymers was executed by a one-step immobilization process involving the immersion of the polymeric surfaces in a water solution containing the peptide at different concentrations. Thereafter, the slides were kept at 70 °C for about 4 h to completely remove the water and to drive the coupling reaction. To validate the success of our immobilization procedure, the test ink was applied, confirming the increase in the surface hydrophilicity of the AMP-functionalized PP slides following the immobilization procedure, due to the introduction of polar groups on the hydrophobic polymer. Moreover, reverse-phase high-precision liquid chromatography (RP-HPLC) analysis was performed to quantify the amount of 1018K6 immobilized on PP surfaces. For this investigation, 1018K6-PP slides, after the coupling reactions, were immersed in distilled water and incubated for 16 h at room temperature under agitation. Then, the polymers were subjected to sonication for 20 min at room temperature and the recovered solutions loaded on an RP-C18 column. By knowing the initial peptide concentrations that were used in the conjugation reaction, the amount of the peptide attached to PP slides was indirectly determined by comparing the peak area in the RP-chromatograms. The data obtained from these analyses showed that the immobilization yield varied from 23%, starting from a peptide concentration of 50 µM, to 5%, when 200 µM was used. The maximum peptide binding (31%) was obtained at 100 µM, which corresponded to a surface coverage of approximately 5.8 nmol/cm^2^, confirming that the initial amount of 1018K6 strongly influenced its binding to synthetic slides (Figure 1). Therefore, 100 μM was selected as the peptide concentration for performing all further experiments.

Concerning the low binding capacity associated with the highest peptide concentration used, it could be attributed to a steric hindrance effect, which limits polymer–peptide interactions and a phenomenon producing water-soluble microaggregates, which can strongly reduce the availability of bioactive molecules for the immobilization reaction.

One of the most important requirements in applying an antimicrobial packaging in the food industries is the stability of the peptide immobilized on the polymers in the conditions of use, because, in this way, it does not require approval as food additive by EFSA (European Food Safety Authority). For this purpose, the slides functionalized with 100 µM 1018K6 were incubated in pure water or in NaCl 1 M at 4 °C for 7 days and the potential release of the peptide from the polymeric support was monitored by RP-HPLC, using the free peptide as a control. Following these analyses, no peptide-release was observed during 7 days of incubation, confirming the strong attachment via the covalent coupling of the bioactive compound, preventing its release from the surface and highlighting the high stability of the system. 

It is worth noting that the projected packaging was reused at least six times in all the subsequent analyses, after washing with EtOH 70% for 1 min, rinsing with water and exposition to UV radiations for 1 h. Surely, this represents an important advantage from the industrial point of view, allowing a substantial decrease in environmental impact based on the concept that “reuse is better than recycling”.

### 3.2. Effects of 1018K6-PP on the Physical Properties and on the Microbiological Quality of Salmon Fillets

It is known that the physicochemical characteristics of raw salmon fillets, such as pH (close to 6) and a high water activity (a_w_), make them highly susceptible to microbial growth, which affects the storability of these products [22]. Therefore, the fish industry is actively seeking methods of preservation to improve quality and marketability of this luxury marine food while economizing on costs. 

**Figure 2:**
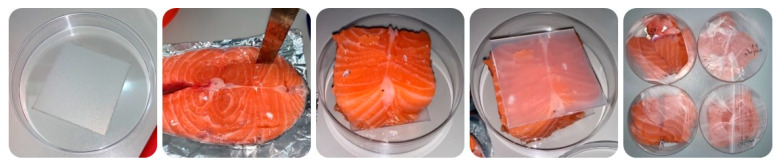
Representative scheme of the experimental preparation of salmon fillets.

To this aim, the antimicrobial effects of 1018K6-PP on the spoilage microbiota and the intrinsic properties of fresh salmon fillets during refrigerated storage were assessed, using the pre-activated and not-functionalized PP slides as control (CTR). In Figure 2, a representative scheme of the different steps applied for the preparation of salmon fillets employed in the microbiological and physicochemical analyses, is shown. 

As reported in Table 1, the initial pH values (pH > 6) for both samples (CTR and 1018K6-PP) were similar to those reported by other authors [23]. Throughout storage, the pH of salmon fillets in contact with the not-functionalized PP slides (CTR) and 1018K6-PP slightly decreased, recording a significant difference between the two groups on the 4th day. This result could be justified by an increase of acid production due to the homogenous proliferation of lactic acid bacteria occurring in these samples during the experimental analysis [24], although Gonzalez-Rodriguez et al. [25] registered an increase of alkalinity in prepacked salmon slices as a result of the ammonia and amines production by bacteria. As far as the water activity is concerned, no significant differences were observed among the experimental groups, with only a minor increase on the fourth day (Table 1). From the microbiological point of view, the initial concentrations of TAB (total aerobic bacteria) at both 30 °C and 7 °C in raw salmons were somewhat higher compared with values reported in previous works [26,27], probably due to poor handling practices during the processing of fish fillets. However, similar data were reported by Wiernasz et al. [28], which referred to a concentration of 4.3 ± 0.2 Log (CFU/g) for total mesophilic bacteria. Indeed, the performed analyses showed that 1018K6-PP samples did not display significant differences (*p* > 0.05) in the growth kinetics of TAB at 30 °C and 7 °C compared with the control samples at the end of the storage period, indicating that the antimicrobial packaging did not have any effect, either positive or negative, on the microbiota of salmon fillets (Table 1). Albeit the total bacterial count represents a key factor in assessing the microbiological quality and safety of foods, it is well known that *Pseudomonas* spp., Enterobacteriaceae and *Brochothrix thermosphacta* are the main microbial family and genera responsible for the off-flavours and the unpleasant odours typical of deteriorating fish and fish products [29]. As far as the evolution of these bacteria is concerned, the samples stored in 1018K6-PP packaging revealed a significant slowdown in the replication of these microorganisms at the 4th day of conservation. Specifically, the sensitivity of bacteria belonging to the Enterobacteriaceae family to antimicrobial activity of the active packaging could make this product interesting for the food industry and promote its applicability as a potential “controller tool” for *Escherichia coli*. Indeed, the inhibitory effect of the innovative packaging was also evident towards beta-glucuronidase-positive *E. coli*, whose levels in treated samples were always below 1.0 Log (CFU/g) in contrast to the CTR [>2.0 Log (CFU/g)]. This finding becomes more relevant when the microbiological limits recommended for *E. coli* (1.0 and 2.7 Log (CFU/g) for minimum and maximum limit, respectively) by the International Commission on Microbiological Specifications for Foods (ICMSF) for the commercialization of fish and fish products [30], are taking into account. Actually, the innovative packaging makes the salmon fillets hygienically suitable throughout the storage period. 

Regarding total coliforms and *Enterococcus faecalis*, the growth curves were very similar for the control and treated groups, but a significant antimicrobial effect of the 1018K6-PP was observed only on the 4th day of storage. Finally, the microbiological results pointed out the ability of the bound peptide to affect the growth of bacteria belonging to *Staphylococcus* genera. Therefore, the antimicrobial coating appears to successfully act on the survival and replicative capacity of this class of microorganisms, showing a significant (*p* < 0.01) difference between the control groups and the treat one on 4th and 7th days. All these findings confirm the results previously obtained with the peptide 1018K6 in a free status [31]. It is worth noting that the same microbiological analyses were performed on salmon fillets packaged in PP films that were not subjected to any surface modification. Interestingly, the obtained results were comparable to those achieved with the pre-activated and not-funzionalized PP films, thus excluding the occurrence of a potential antimicrobial effect determined by the polymeric surfaces activated by plasma alone. It should be pointed out that the microbiological results were not obvious on the basis of two main considerations: (i) the antimicrobial packaging could be unable to kill microbes under conditions of intended use due to the complexity of the fish matrix, which can inactivate the bioactive compound; (ii) 1018K6 could not retain its antimicrobial activity when bound to PP polymers, because the immobilization process could restrict its conformational freedom and influence its orientation, both of which are important features for the peptide activity. 

As far as the potential antimicrobial mechanism, two factors could play a dominant role of the bound 1018K6 with respect to its soluble form:

(1) the high local concentration of the peptide tethered to the polymeric surface;
(2) the strong electrostatic interaction between the cationic peptide chains and anionic bacteria cell membranes (instead of membrane insertion), thus leading to an alteration of the potential across the bacterial membrane, which ultimately triggers cellular death.

To sum up, 1018K6-PPs can be considered a promising instrument to positively affect the quality of perishable products such as fresh salmon based on the microbial effects observed. This suggestion is supported by the important antimicrobial data that the new package exerts against specific spoilage microorganisms responsible for spoilage processes in fish and fish products. However, a potential role of 1018K6-PP in the food safety cannot be excluded, taking into account its action against Enterobacteriaceae and *Staphylococcus* spp. In this scenario, the introduction of 1018K6-PP into the food marketplace could guarantee the availability of safe and natural tools capable of limiting damages of bacterial origin. 

### 3.3. Instrumental Colour Analysis of Salmon Fillets

The colour in fish foods is one of the most important qualities influencing consumer decisions to purchase. Therefore, the impact of 1018K6-PPs on the colour of the packaged salmon fillets was investigated for various storage periods. As reported in Table 2, the lightness (L*) was the only parameter to be influenced significantly by the use of the active packaging, although this phenomenon did not visibly affect the general appearance of the product. Indeed, chroma values are similar in all the samples during the whole experimental period. Our findings are in agreement with Merlo et al. [32], who reported that the use of chitosan film reduces the change in structure of proteins, conferring a darker aspect to treated salmon fillets. This result was justified considering the strong connection between the change in light scattering of the muscle and the variation in lightness. Furthermore, samples packed in 1018K6-PP were found to be slightly more reddish and yellowish (higher values of a* and b*, respectively) than the control ones. According to several authors [32,33,34] the main value taken into account for this fish family is the redness, which is associated to the consumer’s preference and acceptability. Changes in a* value in salmon are due to the addition of carotenoids, such as astaxanthin and cataxanthines, and related to reddish colour of salmonid fishes. However, the scientific community disagrees, and different opinions are reported in literature. Yeşilayer et al. [35] demonstrated that fillets of farmed Atlantic salmons fed with feed containing carotenoids showed high values of yellowness, demonstrating that the typical red-orange colour is represented by both redness and yellowness values. It is worth underling that a* and b* did not differ significantly among all samples by storage time, exhibiting similar ΔE (total colour differences), Δa*, and Δb* values. Therefore, 1018K6-PPs did not produce negative effects on colours parameters, potentially preserving this aspect of salmon samples.

### 3.4. Effect of 1018K6-PP on Chemical Parameters of Salmon Fillets

**Figure 3:**
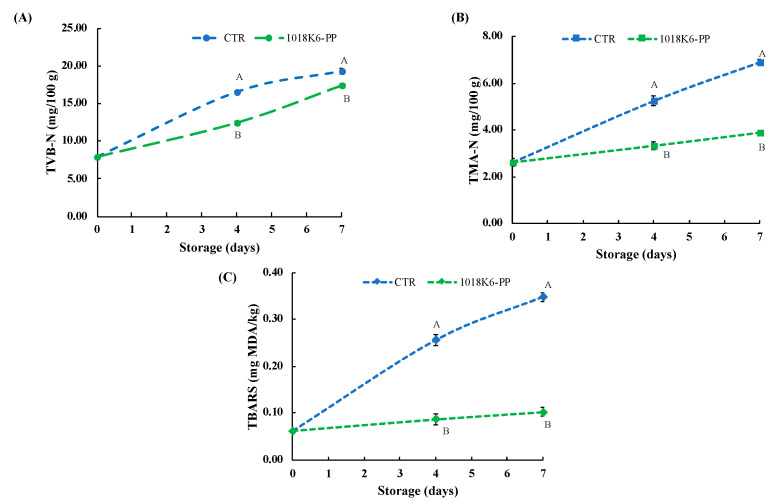
Effects of 1018k6-PP surfaces on the chemical quality of salmon fillets. (**A**) Changes in TVB-N (A), TMA-N (**B**), and TBARS (**C**) of *Salmon salar* fillets packaged in active 1018K6-PP films during storage at 4 °C. CTR (blue lines)—PP films without 1018K6; 1018K6-PP (green lines)—PP films functionalized with 1018K6. Results are means of three independent experiments and error bars represent the standard error (sem). Different letters at each sampling time are used for significantly different samples, according to Tukey test (uppercase letters: *p* < 0.01).

It is common to evaluate the “age” of the food through the study of the microbiological community in order to evaluate the presence and the concentration of specific spoilage microorganisms (SSOs). However, the spoilage of fish and fish products is associated with the occurrence of off-odours due to the production of volatile substances as a result of the bacterial metabolism. Changes in the odour affect the acceptability to consumers, who associate the freshness of fish products to typical organoleptic features. Due to perishable foods being sensitive to variations in appearance, some of the characteristic volatile organic compounds (VOCs) produced by bacteria can be used as potential chemical spoilage indices (CSIs) in fish and fish products [36]. In this study, two chemical quality indicators were used to assess the ability of 1018K6-PP to preserve the quality and sensorial properties, such as the total volatile basic-nitrogen (TVB-N) and trimethylamine-nitrogen (TMA-N) (Figure 3). The choice to detect these two VOCs is dictated by the key role that these chemicals play in the freshness of salmon, being the final products of protein degradation [37].

TVB-N includes the measurement of volatile basic nitrogenous compounds, such as trimethylamine (TMA), dimethylamine (DMA) and other nitrogenic substances, which are produced by bacterial or tissue enzymes from the deamination of amino acids. In the current study, the initial amount of TVB-N in all salmon fillets analysed was 7.89 ± 0.21 mg/100 g (Figure 3A). Significant differences (*p* < 0.01) between salmon samples packaged with CTR-PP and 1018K6-PP slides were observed after 4 days of storage. Specifically, 1018K6-PP appeared, indirectly, to slow down the protein degradation in salmon fillets through the control of microbial growth. Indeed, the great amount of the free amino acids in fish [38,39] are used as substrate by bacteria in their metabolism, with the final production of organic acids, sulphur compounds, ammonia and biogenic amines (BAs) [40,41]. Overall, though 1018K6-PP demonstrated to be efficient in reducing the protein degradation, throughout the entire storage period TVB-N values never reached and overcame the legislative limit of 35 mg/100 g specified by the EU 2019/627 for this fish typology [42]. 

The TMA-N origins by decomposition of trimethylamine N-oxide (TMAO), used from bacteria as a donor of oxygen molecules in their respiratory metabolism in fish and fish products stored at refrigeration temperature [43,44,45]. Due to the importance of the initial amount of TMAO in the muscle, the concentration of TMA-N is strongly related to the species of fish, and *Salmo salar* is naturally rich in trimethylamine N-oxide [46]. In Figure 3B, the trends of TMA-N over time are displayed. Specifically, the samples packed with 1018K6-PPs showed the lowest TMA-N values (*p* < 0.01) at both 4 and 7 days of storage, in agreement with the above reported values of TVB-N, thus reinforcing the hypothesis that active slides affect the spoilage microbial communities. In fact, it is well-known that the TMA production is mainly operated by bacteria belonging to the Enterobacteriaceae family, which, the results showed, proved sensitive to the antimicrobial activity of the bound peptide, as already reported in Table 1 [47,48]. Although TMA is considered a good indicator of the deterioration progress, no maximum legislative limits for TMA concentrations were defined and different values were proposed. However, according to Shumilina et al. [49], who reported 4.2 mg/100 g as the acceptability limit for fish, the freshness was preserved only in salmon fillets put in contact with 1018K6-PP (TMA < 5 mg/100 g).

Finally, measurements of thiobarbituric acid reactive substances (TBARS) expressed as malonyldialdehyde (MDA) levels, were performed in order to investigate lipid oxidation, which is a very important event determining the quality of foods, especially of those containing highly unsaturated fats, such as fish [50,51]. As shown in Figure 3C, the TBARS values in control fillets increased significantly during refrigerated storage in contrast to that observed in the packaged fillets with 1018K6-PP slides. Therefore, 1018K6-PP is able to exert antioxidant properties, but this finding is not surprising given the well-known correlation between lipid oxidation and bacterial contamination [52]. 

Indeed, MDA is the main aldehyde produced as a result of the decomposition of unsaturated fatty acids—also a bacterial operation—thus, remarking on the antimicrobial efficacy of the active films. The chemical analyses performed on the salmon fillets packaged in unmodified PP films demonstrated that the plasma activation by itself was not able to allow the polymers to affect the quality of these fish products. 

Overall, the comprehensive analyses of microbiological and chemical parameters pointed out two main aspects: the key roles of TVB-N, TMA and MDA as chemical spoilage indices in perishable food and the effectiveness of 1018K6-PP in preserving salmon fillets. As reported by Prabhakar et al. [53], the assumption of the interconnection among bacterial concentrations and chemical metabolites production is already consolidated, as is the link between TVB-N/TMA levels and quality. Therefore, our findings confirmed this strong link, and the candidate 1018K6-PP as a valuable packaging technology capable of guaranteeing longer durability for highly perishable foods, such as raw salmon.

### 3.5. Panelists’ Sensory Evaluation

**Figure 4:**
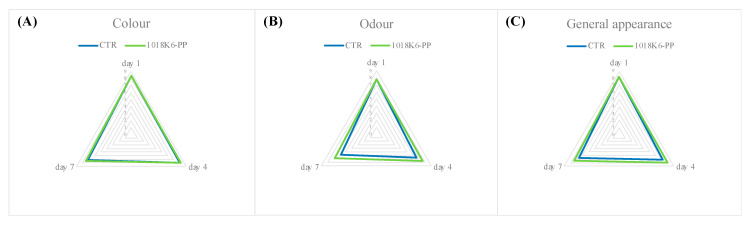
Changes in colour (**A**), odour (**B**), and overall appearance (**C**) of salmon fillets during storage period. CTR (blue lines)—PP films without 1018K6; 1018K6-PP (green lines)—PP films functionalized with 1018K6.

Sensory perception is the tool through which the consumers choose foods at a store, trusting in their senses and adopting an immediate and easy system for evaluating freshness and quality [54]. In this study, the organoleptic features appeared to be partially influenced by the packaging technology used. As reported in Figure 4, the representation of observed sensory characteristics highlighted an important consequence of the use of antimicrobial slides on the production of off-odours. Despite the initial good quality of all samples, the salmon fillets belonging to the control groups showed signs of spoilage as early as the 4th day of storage at refrigeration temperature. The judges rated the control samples as “poor freshness quality” products, due to the score of odour and of the general appearance obtained by the end of the trial. Contrarily, the treated samples maintained good sensory characteristics over time. In agreement with those reported above for VOCs, the demonstrated antimicrobial activity (Table 1) of 1018K6-PP seems to indirectly control the negative changes in the chemical structure and metabolites production of salmon fillets occurring during storage [55,56]. Furthermore, the scores of overall appearances of treated samples pointed out the absence of negative influences of the novel active packaging on the sensory features, due to the colourless and odourless nature of the 1018K6 molecules.

### 3.6. Microbial Challenge Testing of L. monocytogenes on Salmon Fillets Packaged with 1018K6-PP

Foodborne diseases are a reality affecting thousands of people in industrialized countries every year. Amongst the bacterial pathogens responsible of severe human toxi-infections*, Listeria monocytogenes* is considered one of the most dangerous. Due to their origin and the way in which they are processed, fish products show an increased incidence rate of listeriosis, and then they represent typical food vehicles of high levels of microbiological contamination, taking into account that this bacterium is able to grow also at refrigeration temperatures. Therefore, challenge testing of the food products with *L. monocytogenes* is recommended to assess the potential for growth, both qualitatively and quantitatively, in the foods at risk.

**Figure 5:**
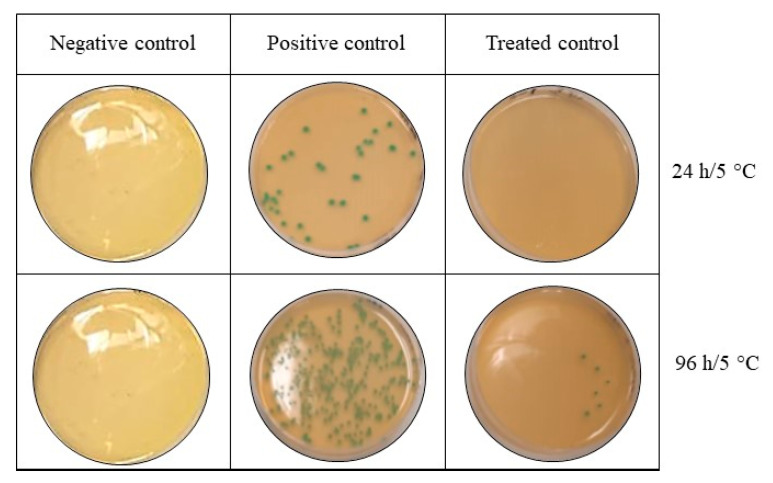
Bactericidal activity of polymer functionalized with 1018K6 against *L. monocytogenes* on salmon fillets. Negative control—untreated salmon fillets; positive control—salmon fillets treated with not-functionalized PP; treated control—salmon fillets treated with 1018K6-PP.

In this context, the anti-listerial efficacy of 1018K6-PP was evaluated in salmon fillets stored at 5 °C for 96 h (Figure 5). In order to confer greater confidence in the assessment of the likelihood of a particular strain to compromise food safety, mixed cultures of three *L. monocytogenes* strains isolated from fish were used at a concentration of ca. 150 CFU/mL. This value of inoculum is representative for the natural contamination of *L. monocytogenes* commonly encountered in fresh foods, taking into account that 100 CFU/mL is the threshold limit considered as low risk for causing listeriosis. In addition, the use of food isolates is recommended because it is likely to represent better the behaviour of naturally contaminating strains.

The results of the challenge test performed on salmon fillets indicated that the antimicrobial packaging was effective in inhibiting the growth and survival of the pathogen on the surface of the fresh food during storage in contrast to the untreated control. Indeed, a complete inhibition of *L. monocytogenes* was observed after 72 h incubation, with a slight decrease (95%) at the end of the assay (96 h), thus suggesting that our system could be used to preserve the safety of fish products during storage.

Overall, our results show the positive impact of the effectiveness of 1018K6-PP packaging on food safety when the target microorganism is a foodborne pathogen of great present concern, such as *L*. *monocytogenes*.

### 3.7. Evaluation of 1018K6-PPs Slides on the Physicochemical, Microbial and Sensorial Properties of Sarda Sarda Burgers

**Figure 6:**
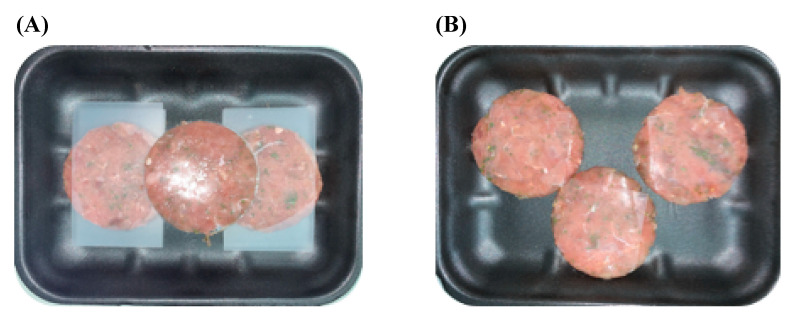
Representative scheme of preparation of *Sarda sarda* burgers employed in the microbiological and physico-chemical analyses. (**A**) Burgers of *Sarda sarda* treated with PPs films not-functionalized with 1018K6 (CTR); (**B**) burgers of *Sarda sarda* treated with PPs films functionalized with 1018K6 (1018K6-PP).

In order to evaluate the versatility of our active packaging, a different typology of food matrices was included in the experimental design. To this aim, microbiological, physicochemical and sensorial analyses were performed on fish burgers of bonito (*Sarda sarda)* packaged with 1018K6-PP slides. This analysis was aimed at also verifying the effectiveness of 1018K6-PP against minced fish meat, which is notoriously characterized by higher level of microorganisms than fillets because of the shredding process underlying their manufacture [57]. The scheme used to set up the *Sarda sarda* hamburger employed in the experimental trials, is shown in Figure 6. 

As reported in Table 3, the initial amounts of TAB were significantly different between salmon fillets (Table 1) and fish burgers (Table 3), in which more than 1 Log (CFU/g) of mesophilic bacteria were enumerated. For this reason, fish burgers represent a difficult challenge. Regarding antimicrobial activity, 1018K6-PPs negatively affected the growth of specific microorganisms, including the total bacterial count. During the storage, the mesophilic TAB increased significantly in control samples, until reaching a concentration greater than 8 Log (CFU/g) by the 7th day, in contrast to that observed in the samples packaged with the antimicrobial slides, in which the maximum acceptable limit set by ICMSF for TAB [7 Log (CFU/g)] was never exceeded during 7 days of storage. Furthermore, due to the key role of mesophilic bacteria in the production of metabolites and off-odours, the antimicrobial activity of 1018K6-PP produced a beneficial effect on the overall appearance of fish burgers and their chemical profile. Therefore, our findings not only confirmed the effectiveness of the new package in slowing the growth of the same bacterial communities described for salmon fillets but also the obtained results enhanced the potential role of 1018K6-PP as a tool for monitoring microbiologic contaminations. 

It is worth noting that the same analyses were performed on bonito burgers packaged in PP films and not subjected to any surface modification and no discrepancy in the results was observed with respect to those obtained with the pre-activated PP films alone.

As far as the determination of colour values, the changes in this parameter in fish burgers over time overlapped the data collected for salmon fillets (Table 4). Specifically, the samples belonging to the control group appeared less dark than the others, affirming the hypothesis of an increase in proteolysis. Moreover, no differences were highlighted among samples in a* and b* values and, consequently, in total colour differences (ΔE), variations in a* (Δa*), and in b* (Δb*). 

**Figure 7:**
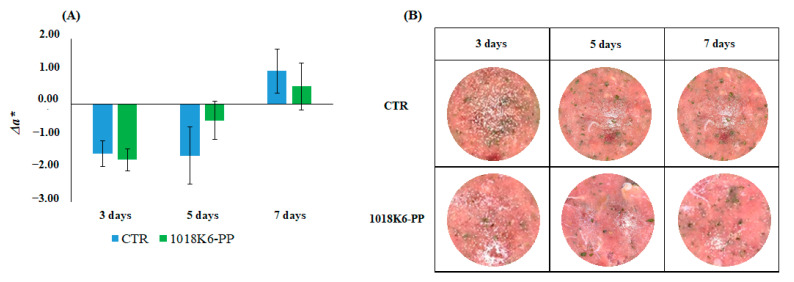
(**A**) Analysis of a* variation (Δa*) in bonito fish burgers during the storage period. Results are means of three independent experiments and error bars represent the standard error (sem). CTR (blue)—PP films without 1018K6; 1018K6-PP (green)—PP films functionalized with 1018K6. (**B**) Photos of *Sarda sarda* burgers packaged with the control and functionalized slides at each sampling time.

The graph reported in Figure 7 showed the positive effect of the active packaging on the redness on 3rd and 7th day, although slightly. 

**Figure 8:**
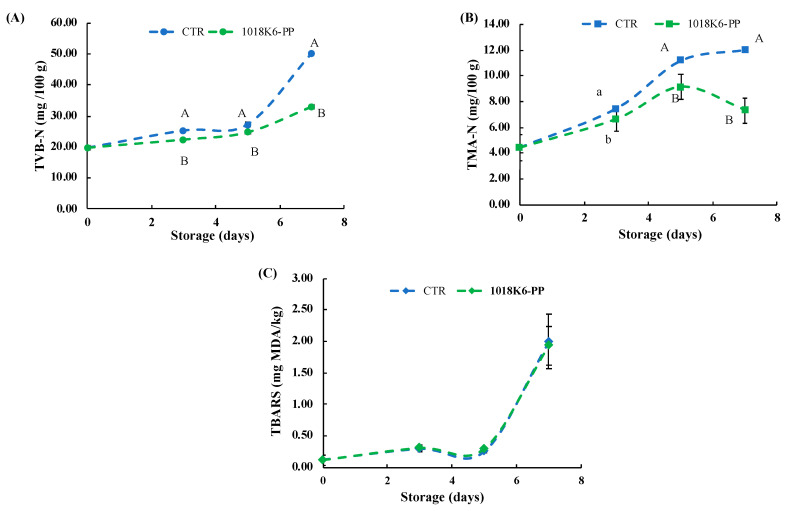
Effects of 1018K6-PP slides on the chemical quality of *Sarda sarda* burgers. Changes in TVB-N (**A**), TMA-N (**B**) and TBARS (**C**) of *Sarda sarda* burgers packaged in active 1018K6-PP films during storage at 4 °C. CTR (blue lines)—PP films without 1018K6; 1018K6-PP (green lines)—PP films functionalized with 1018K6. Results are means of three independent experiments and error bars represent the standard error (sem). Different letters at each sampling time are used for significantly different samples, according to Tukey test (uppercase letters: *p* < 0.01; lowercase letters: *p* < 0.05).

Moreover, the experimentation on fish burgers marked the important contribution of the antimicrobial molecule in slowing down the protein degradation. Indeed, significant differences were found among samples packed in active films and control ones and the gap recorded between the corresponding TMA-N and TVB-N values proved the concrete beneficial effect of the 1018K6-PP (Figure 8). 

**Figure 9:**
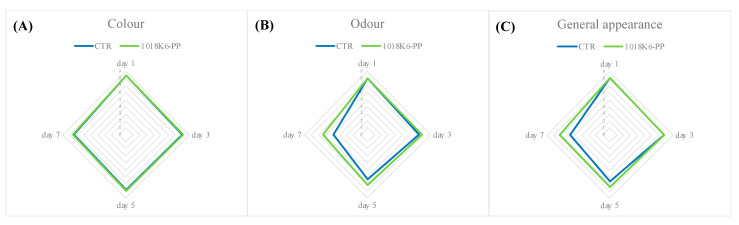
Changes in colour (**A**), odour (**B**), and overall appearance (**C**) of bonito fish burgers during storage period. CTR (blue lines)—PP films without 1018K6; 1018K6-PP (green lines)—PP films functionalized with 1018K6.

Finally, the off-odours drastically affected the judgments (Figure 9), by which the control samples were labelled as unpleasing foods, probably due to their content in TVB-N and TMA-N. This result was expected considering that the odour weight on the panellists’ choices is the most critical sensory characteristic for fish products [58].

Finally, our findings allowed also supposing a positive effect of 1018K6-PP on the quality parameters of bonito burgers, considering the significant differences between the two experimental groups in microorganisms concentrations and CSIs levels, which strongly affected on the sensory appearance of samples. The off-odours and the changes in lightness were demonstrated to be the main visible properties associated to the spoilage processes, so that they could be considered alarm bells for the consumers.

## 4. Conclusions

As stated, fish is a highly perishable food characterized by a short shelf-life. Refrigeration is probably one of the most used methods for fish preservation, but several deteriorative quality changes occur during storage, particularly in texture, colour and flavour, limiting shelf-life. Therefore, there is an important and urgent need to find alternative strategies to overcome existing challenges that are associated with fish spoilage, which will ultimately benefit both the producers and consumers. In the present study, two different kinds of fish foods, *Salmon salar* fillets and *Sarda sarda* burgers, were used to obtain information about the feasibility of the potential application of 1018K6-PP packaging in the food industry. The results showed that 1018K6-PP helped to maintain the chemical and microbial quality of this kind of product without inducing sensory alterations during refrigerated storage. Therefore, the antimicrobial packaging used in the present study represents an excellent and promising option for the preservation of fish foods due to its antimicrobial, non-toxic and re-usability properties, and thereby reduce the occurrence of foodborne illness.

## Figures and Tables

**Table 1:** Evaluation of microbiological counts [Log (CFU/g)] in salmon fillets packaged in active PP films functionalized with 1018K6 by storage time.

		*Day*	0	4	7
			*m ± sem*	*m ± sem*	*m ± sem*
TAB 30 °C	CTR		4.76 ± 0.06 ^A^	6.77 ± 0.07 ^B^	6.89 ± 0.02 ^B^
	1018K6-PP		4.76 ± 0.06 ^A^	6.73 ± 0.01 ^B^	6.88 ± 0.01 ^C^
TAB 7 °C	CTR		2.91 ± 0.04 ^A^	4.24 ± 0.01 ^B,X^	5.32 ± 0.04 ^C^
	1018K6-PP		2.91 ± 0.04 ^A^	4.56 ± 0.04 ^B,Y^	5.28 ± 0.04 ^C^
Coliforms	CTR		1.91 ± 0.05 ^A^	3.91 ± 0.04 ^a,B,X^	3.44 ± 0.19 ^b,B^
	1018K6-PP		1.91 ± 0.05 ^A^	3.56 ± 0.07 ^B,Y^	3.44 ± 0.18 ^B^
Enterobacteriaceae	CTR		0.96 ± 0.01 ^A^	3.86 ± 0.07 ^B,x^	3.07 ± 0.04 ^C,X^
	1018K6-PP		0.96 ± 0.01 ^A^	3.66 ± 0.04 ^B,y^	3.96 ± 0.04 ^C,Y^
*Pseudomonas* spp.	CTR		4.31 ± 0.09 ^A^	7.32 ± 0.10 ^B,X^	7.44 ± 0.16 ^B,X^
	1018K6-PP		4.31 ± 0.09 ^A^	6.28 ± 0.05 ^B,Y^	6.91 ± 0.04 ^C,Y^
*E. coli*	CTR		*ni* ^A^	2.07 ± 0.09 ^B,X^	2.95 ± 0.03 ^C,X^
	1018K6-PP		*ni*	*ni* ^Y^	*ni* ^Y^
*Enterococcus faecalis*	CTR		3.32 ± 0.08 ^A^	3.96 ± 0.01 ^B,X^	5.07 ± 0.12 ^C,x^
	1018K6-PP		3.32 ± 0.08 ^a,A^	2.96 ± 0.12 ^bA,Y^	4.74 ± 0.06 ^B,y^
*B. thermosphacta*	CTR		4.98 ± 0.07 ^A^	5.98 ± 0.03 ^B,X^	7.36 ± 0.14 ^C,X^
	1018K6-PP		4.98 ± 0.07 ^A^	6.81 ± 0.04 ^B,Y^	5.96 ± 0.19 ^C,Y^
*Staph*. coagulase positive	CTR		*ni* ^A^	2.26 ± 0.09 ^B,X^	3.19 ± 0.05 ^C,X^
	1018K6-PP		*ni* ^A^	*ni* ^A,Y^	1.96 ± 0.02 ^B,Y^
pH	CTR		6.25 ± 0.01 ^A^	6.18 ± 0.02 ^B,X^	6.07 ± 0.03 ^C^
	1018K6-PP		6.25 ± 0.01 ^A^	6.11 ± 0.01 ^B,Y^	6.02 ± 0.01 ^C^
a_w_	CTR		0.973 ± 0.003 ^a^	0.981 ± 0.001 ^b^	0.982 ± 0.002 ^b^
	1018K6-PP		0.973 ± 0.003 ^a^	0.979 ± 0.000 ^b^	0.980 ± 0.002 ^b^

*ni*: not isolated. In each storage day, three samples by experimental group were analysed. Statistical analysis was performed comparing experimental groups at each sampling time and within each experimental group along the ripening period. All data were presented as mean (m) ± standard error (sem). Different superscript uppercase letters indicate a significant difference at *p* < 0.01. Different superscript lowercase letters indicate a significant difference at *p* < 0.05. ^a–c^ In the same row mean values (same group in different days) followed by different letters show significant differences. ^x,y^ In the same column mean values (different groups on the same sampling time) followed by different letters show significant differences.

**Table 2:** Changes in colour indices of the salmon fillets packaged in active PP films functionalized with 1018K6 by storage time.

		*Day*	0	4	7
			*m ± sem*	*m ± sem*	*m ± sem*
L*	CTR		43.51 ± 1.47 ^a^	45.93 ± 0.63 ^a,X^	48.23 ± 0.67 ^b,x^
	1018K6-PP		43.51 ± 1.47 ^A^	36.13 ± 1.89 ^B,Y^	44.48 ± 1.51 ^A,y^
a*	CTR		16.78 ± 0.83	19.75 ± 1.41	15.94 ± 1.44
	1018K6-PP		16.78 ± 0.83	20.02 ± 1.25 ^a^	16.63 ± 0.59 ^b^
b*	CTR		21.15 ± 1.68	23.55 ± 2.86	15.94 ± 1.99
	1018K6-PP		21.15 ± 1.68	24.40 ± 3.40	17.01 ± 1.62
*Chroma*	CTR		27.01 ± 1.82	30.76 ± 3.06	22.55 ± 2.43
	1018K6-PP		27.01 ± 1.82	31.63 ± 3.30	23.82 ± 1.52
*Hue angle*	CTR		51.46 ± 1.01 ^A^	49.75 ± 1.64 ^a^	44.80 ± 0.92 ^b,B^
	1018K6-PP		51.46 ± 1.01 ^a^	50.10 ± 2.92	45.42 ± 1.97 ^b^
Δ*E*	CTR			6.31 ± 2.26	7.22 ± 0.88
	1018K6-PP			9.47 ± 1.45	6.45 ± 1.82
Δa*	CTR			2.97 ± 1.94	−0.83 ± 0.61
	1018K6-PP			3.24 ± 1.53	−0.14 ± 1.21
Δb*	CTR			2.40 ± 3.14 ^a^	−5.21 ± 0.67 ^b^
	1018K6-PP			3.24 ± 2.55	−4.15 ± 3.28

On each sampling day, three samples by experimental group were analysed. Statistical analysis was performed comparing experimental groups at each sampling time and within each experimental group along the ripening period. All data were presented as mean (m) ± standard error (sem). Different superscript uppercase letters indicate a significant difference at *p* < 0.01. Different superscript lowercase letters indicate a significant difference at *p* < 0.05. ^a,b^ In the same row mean values (same group in different days) followed by different letters show significant differences. ^x,y^ In the same column mean values (different groups on the same sampling time) followed by different letters show significant differences.

**Table 3:** Evaluation of microbiological counts (Log CFU/g) in *Sarda sarda* burgers packaged in active PP films functionalized with 1018K6 by storage time.

		*Day*	0	3	5	7
			*m ± sem*	*m ± sem*	*m ± sem*	*m ± sem*
TAB 30 °C	CTR		6.25 ± 0.02 ^a,A^	6.52 ± 0.17 ^A^	6.74 ± 0.17 ^b,A^	8.14 ± 0.08 ^X,B^
	1018K6-PP		6.25 ± 0.02	6.17 ± 0.12	6.22 ± 0.22	6.37 ± 0.34 ^Y^
TAB 7 °C	CTR		5.16 ± 0.09 ^A^	6.36 ± 0.08 ^B^	6.79 ± 0.07 ^C,X^	8.17 ± 0.04 ^D,X^
	1018K6-PP		5.16 ± 0.09 ^A^	6.01 ± 0.16 ^B^	5.94 ± 0.19 ^B,Y^	6.50 ± 0.39 ^B,Y^
Coliforms	CTR		4.61 ± 0.02 ^A^	5.39 ± 0.09 ^a,B,X^	4.90 ± 0.04 ^C^	5.15 ± 0.06 ^b,B,X^
	1018K6-PP		4.61 ± 0.02	4.60 ± 0.23 ^Y^	4.62 ± 0.16	4.35 ± 0.25 ^Y^
Enterobacteriaceae	CTR		3.26 ± 0.17 ^A^	5.96 ± 0.57 ^B,C,X^	4.98 ± 0.10 ^B^	5.26 ± 0.00 ^C^
	1018K6-PP		3.26 ± 0.17 ^A^	3.63 ± 0.46 ^a,A,Y^	4.97 ± 0.16 ^b,B^	5.11 ± 0.15 ^B^
*Pseudomonas* spp.	CTR		5.91 ± 0.02 ^a,A^	6.51 ± 0.20 ^bB,x^	5.79 ± 0.10 ^A,x^	8.59 ± 0.09 ^C,X^
	1018K6-PP		5.91 ± 0.02 ^A^	5.91 ± 0.13 ^y^	5.47 ± 0.08 ^B,y^	6.44 ± 0.36 ^Y^
*E. coli*	CTR		1.50 ± 0.12 ^a^	1.80 ± 0.20 ^A^	1.62 ± 0.11 ^A^	1.11 ± 0.09 ^b,B^
	1018K6-PP		1.50 ± 0.12	1.32 ± 0.18	1.19 ± 0.23	1.28 ± 0.16
*Enterococcus faecalis*	CTR		4.39 ± 0.13 ^A^	4.41 ± 0.09 ^A,X^	3.21 ± 0.23 ^B^	3.96 ± 0.00 ^C^
	1018K6-PP		4.39 ± 0.13 ^a,A^	3.39 ± 0.13 ^B,Y^	3.81 ± 0.39	3.87 ± 0.21 ^b^
*B. thermosphacta*	CTR		*ni* ^A^	*ni* ^A^	1.98 ± 0.00 ^B,X^	1.98 ± 0.00 ^B,X^
	1018K6-PP		*ni*	*ni*	*ni* ^Y^	*ni* ^Y^
*Staph.* coagulase positive	CTR		4.45 ± 0.01 ^A^	5.64 ± 0.08 ^B,X^	4.23 ± 0.14 ^A,X^	4.37 ± 0.26 ^A,X^
	1018K6-PP		4.45 ± 0.01 ^A^	4.12 ± 0.16 ^a,A,Y^	3.6 ± 0.08 ^b,B,Y^	3.19 ± 0.22 ^B,Y^
pH	CTR		6.20 ± 0.01 ^A^	6.18 ± 0.00 ^A,x^	6.24 ± 0.01 ^B^	6.39 ± 0.03 ^C,X^
	1018K6-PP		6.20 ± 0.01 ^a^	6.21 ± 0.01 ^y^	6.23 ± 0.00 ^b^	6.26 ± 0.03 ^b,Y^
a_w_	CTR		0.976 ± 0.006	0.969 ± 0.001 ^a^	0.972 ± 0.001	0.974 ± 0.001 ^b^
	1018K6-PP		0.976 ± 0.006	0.963 ± 0.009	0.973 ± 0.000	0.974 ± 0.002

*ni*—not isolated. In each sampling day, three samples were analysed by experimental group. Statistical analysis was performed comparing experimental groups at each sampling time and within each experimental group along the ripening period. All data were presented as mean (m) ± standard error (sem). Different superscript uppercase letters indicate a significant difference at *p* < 0.01. Different superscript lowercase letters indicate a significant difference at *p* < 0.05. ^a–d^ In the same row mean values (same group in different days) followed by different letters show significant differences. ^x,y^ In the same column mean values (different groups on the same sampling time) followed by different letters show significant differences.

**Table 4:** Changes in colour indices of *Sarda sarda* burgers packaged in active PP films functionalized with 1018K6 by storage time.

		*Day*	0	3	5	7
			*m ± sem*	*m ± sem*	*m ± sem*	*m ± sem*
L*	CTR		42.68 ± 1.09	41.07 ± 0.51 ^A^	42.95 ± 1.20	45.04 ± 0.77 ^B,x^
	1018K6-PP		42.68 ± 1.09 ^a^	39.76 ± 0.74 ^b,A^	42.32 ± 0.80 ^a^	42.79 ± 0.70 ^B,y^
a*	CTR		6.12 ± 0.89	4.93 ± 0.20 ^A^	4.87 ± 0.47 ^A,x^	7.46 ± 0.27 ^B,X^
	1018K6-PP		6.79 ± 0.34 ^a,A^	4.74 ± 0.37 ^B^	5.95 ± 0.20 ^b,A,y^	6.12 ± 0.63 ^Y^
b*	CTR		12.42 ± 0.38 ^a,A^	15.61 ± 0.31 ^B,x^	10.71 ± 0.77 ^A^	10.93 ± 0.60 ^b,A^
	1018K6-PP		12.42 ± 0.38 ^a,A^	14.50 ± 0.37 ^B,y^	12.29 ± 0.38 ^a,A^	10.53 ± 0.70 ^b,A^
*Chroma*	CTR		13.89 ± 0.44 ^a,A^	16.37 ± 0.33 ^B,x^	11.79 ± 0.83 ^b,A,x^	13.24 ± 0.61 ^A^
	1018K6-PP		14.15 ± 0.43	15.28 ± 0.35 ^A,y^	13.66 ± 0.37 ^B,y^	12.21 ± 0.86 ^B^
*Hue angle*	CTR		63.86 ± 3.54 ^a^	72.47 ± 0.62 ^b,A^	65.48 ± 1.87 ^B^	55.51 ± 1.09 ^b,B,x^
	1018K6-PP		61.34 ± 1.08 ^A^	71.86 ± 1.45 ^B^	64.11 ± 0.97 ^A^	60.06 ± 1.83 ^A,y^
Δ*E*	CTR			4.20 ± 0.60 ^A^	3.61 ± 0.88 ^B^	4.06 ± 0.91 ^B^
	1018K6-PP			4.60 ± 0.80 ^A^	2.79 ± 0.70 ^a,B^	3.65 ± 0.76 ^b,B^
Δb*	CTR			3.10 ± 0.00	−1.71 ± 0.91	−1.49 ± 0.54
	1018K6-PP			2.09 ± 0.39	−0.13 ± 0.37	−1.88 ± 0.75

In each sampling day, three samples were analysed by experimental group. Statistical analysis was performed comparing experimental groups at each sampling time and within each experimental group along the ripening period. All data were presented as mean (m) ± standard error (sem). Different superscript uppercase letters indicate a significant difference at *p* < 0.01. Different superscript lowercase letters indicate a significant difference at *p* < 0.05. ^a,b^ In the same row mean values (same group in different days) followed by different letters show significant differences. ^x,y^ In the same column mean values (different groups on the same sampling time) followed by different letters show significant differences.

## Data Availability

Data is contained within the article.
